# Extensive subclinical sinusitis leading to *Moraxella osloensis* meningitis

**DOI:** 10.1016/j.idcr.2016.08.007

**Published:** 2016-09-21

**Authors:** A. Fox-Lewis, G. Coltart, S. Rice, R. Sen, Y. Gourtsoyannis, H. Hyare, R.K. Gupta

**Affiliations:** a Division of Infection, University College London Hospital NHS Foundation Trust, London, UK; b Radiology Department, University College London Hospital NHS Foundation Trust, London, UK; c Department of Infection, University College London, London, UK

## Abstract

We report a case of a 31 year old male with extensive subclinical sinusitis leading to erosion in the cribriform plate and subsequent meningitis caused by the organism *Moraxella osloensis*. The patient presented to the emergency department with rapid onset confusion, neck stiffness and headache. Inflammatory markers, renal and liver function, and a chest radiograph were all normal. CT Head showed extensive polyp disease in the paranasal sinuses with expansion of the left frontal sinus and CT Sinuses revealed an area of low attenuation in the cribriform plate consistent with bony erosion. MRI Head showed thick loculated sinus inflammation. Lumbar puncture yielded CSF with a high white cell count of predominantly mononuclear cells, no visible organisms and an elevated protein. CSF microscopy, culture and viral PCR were not diagnostic, and so the CSF was sent for 16S rDNA PCR screening, which identified the rDNA of *Moraxella osloensis*. *Moraxella osloensis* is a rare cause of bacterial meningitis, with only a few reported cases. This case illustrates that sinusitis, while a common condition, when severe can predispose to intracranial infection with atypical and low virulence organisms such as *Moraxella* species, which do not commonly cause invasive CNS disease. This case represents the first case of *Moraxella osloensis* meningitis reported from the United Kingdom.

## Introduction

*Moraxella osloensis* is part of the normal flora of the human respiratory tract, and is one of the seven species of the Moraxella genus, along with *M. atlantae*, *M. canis*, *M. catarrhalis*, *M. lacunata*, *M. lincolnii* and *M. nonliquefaciens* [1]. Members of the genus *Moraxella* are oxidase-positive, non-motile, and asaccharolytic coccobacilli. Infections attributed to *Moraxella osloensis* include endocarditis, osteomyelitis, septic arthritis, vaginitis, bacteremia, and rarely, meningitis [2].

Meningitis makes up 10% of the intracranial complications of sinusitis in paediatric populations [3], however the presence of sinusitis is a predictor of a more favorable outcome [4]. We report a case of extensive subclinical sinusitis leading to erosion in the cribriform plate with subsequent meningitis caused by the organism *Moraxella osloensis*. To our knowledge there are only seven previously reported cases of meningitis caused by *Moraxella osloensis*: one case from the Netherlands [5]; one from Scandinavia [6]; two from Germany [7], [8] and three cases from Korea [9]. Two of these cases had predisposing factors, one patient with a CSF shunt in situ, and one with C8 compliment deficiency, while the remaining cases were in immunocompetent individuals. This case represents the first case of *Moraxella osloensis* meningitis reported from the United Kingdom.

## Case report

A 31 year old male with no significant past medical history presented to the emergency department with rapid onset confusion, neck stiffness and headache. There was no history of recent travel outside the UK, and no drug use or alcohol intake. Initial assessment revealed normal vital signs and an unremarkable physical examination with no focal neurology. There was no neck stiffness or photophobia. Abbreviated mental test score was 6/10 and Glasgow Coma Score was 13/15. The patient was agitated, confused and disoriented in time and place. Inflammatory markers, renal and liver function, and a chest radiograph were all normal.

**Fig. 1:**
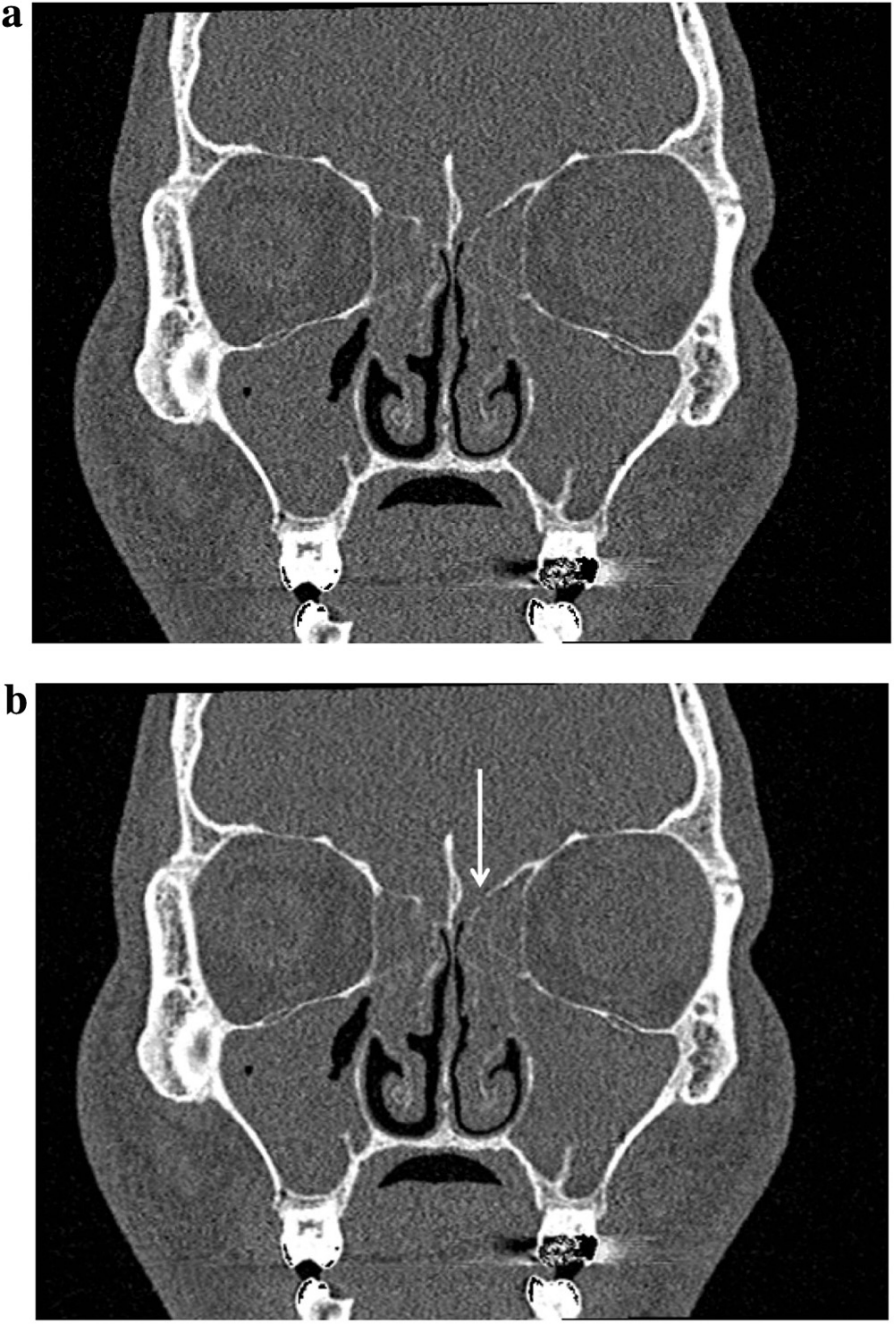
(a) CT Sinuses showing area of bony erosion in the left aspect of the cribriform plate (unmarked). (b) CT Sinuses showing area of bony erosion in the left aspect of the cribriform plate (marked).

**Fig. 2:**
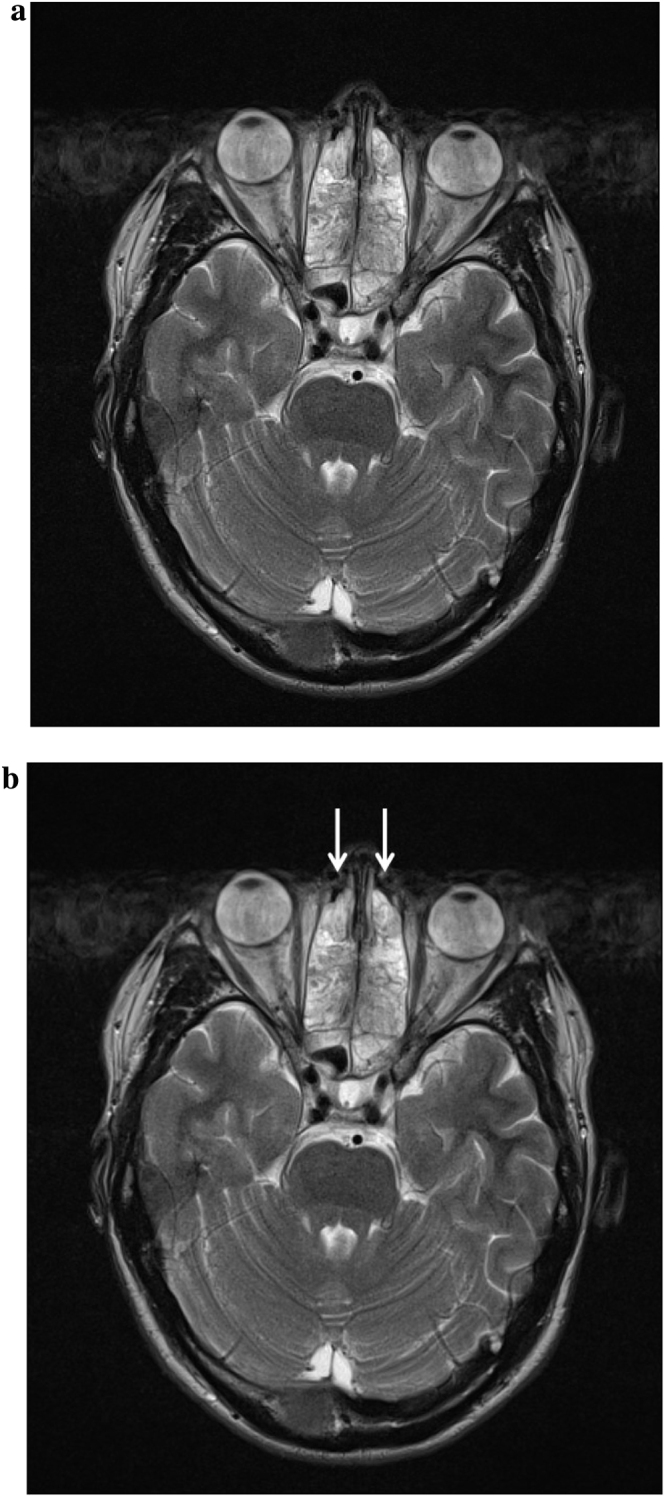
(a) MRI Head (T2 weighted) showing severe ethmoid sinusitis (unmarked). (b) MRI Head (T2 weighted) showing severe ethmoid sinusitis (marked).

**Fig. 3:**
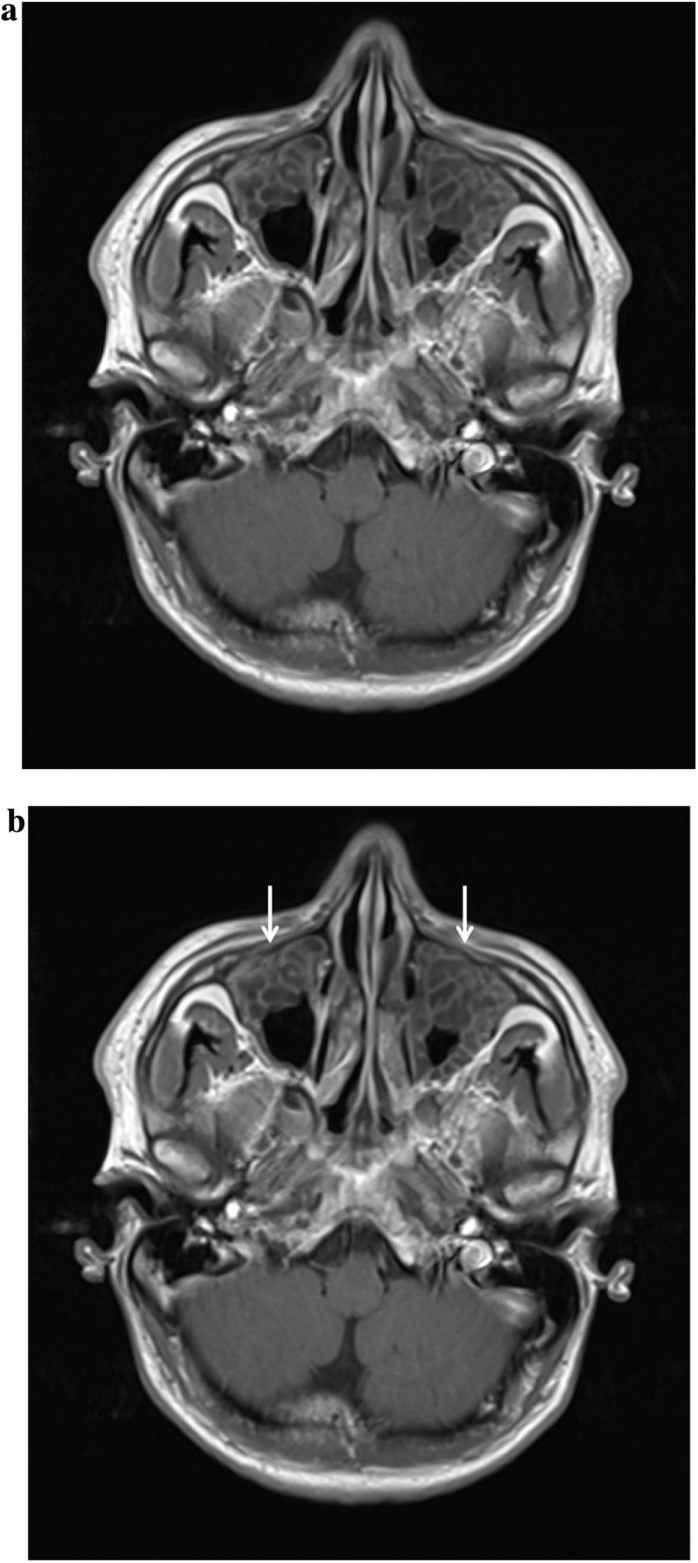
(a) MRI Head (T1 weighted with contrast) showing extensive loculated sinusitis (unmarked). (b) MRI Head (T1 weighted with contrast) showing extensive loculated sinusitis (marked).

**Fig. 4:**
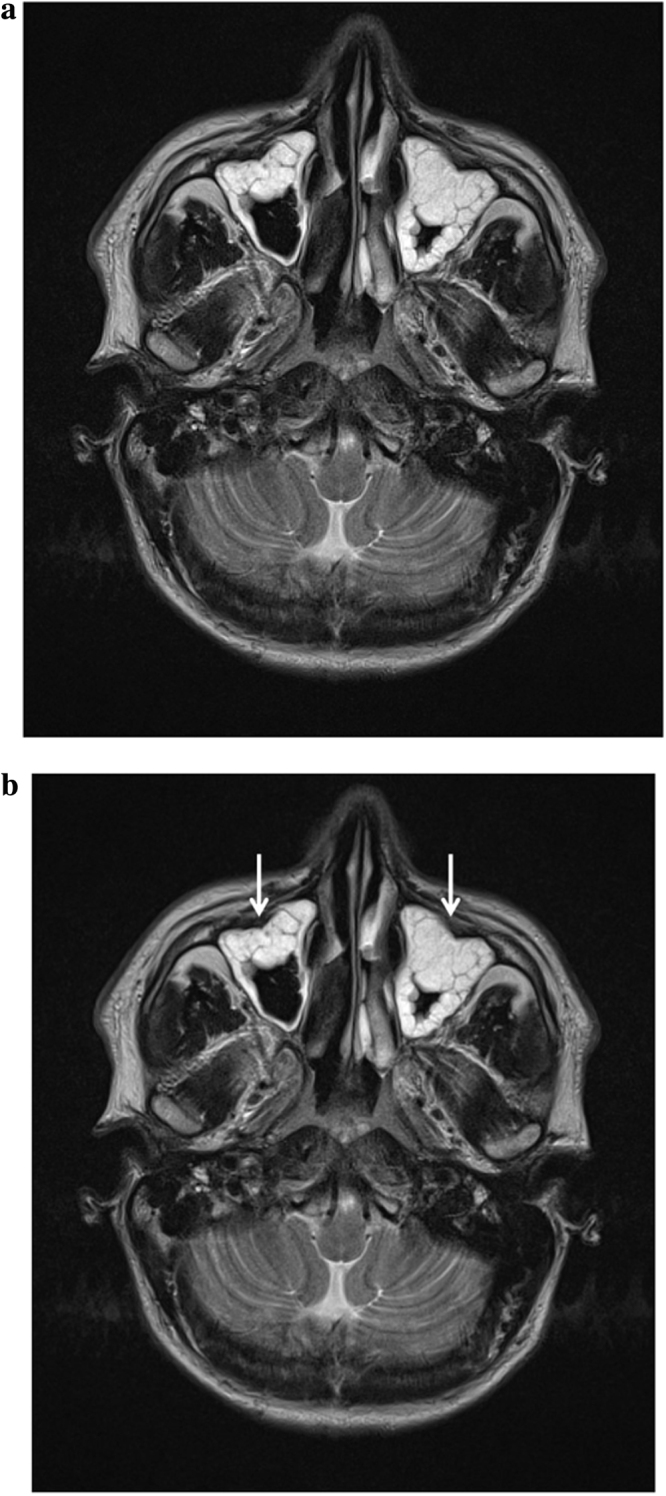
(a) MRI Head (T2 weighted) showing extensive loculated sinusitis (unmarked). (b) MRI Head (T2 weighted) showing extensive loculated sinusitis (unmarked).

CT Head showed extensive polyp disease in the paranasal sinuses with expansion of the left frontal sinus. ENT review found mild erythema only in the oropharynx, with erythematous and rhinitic polypoid mucosa in the nasal cavity. A subsequent CT Sinuses revealed an area of low attenuation in the left aspect of the cribriform plate consistent with bony erosion (Fig. 1a and b). MRI Head showed thick loculated sinus inflammation (Figs. 2a–4b ). Lumbar puncture yielded colorless CSF with an opening pressure of 18 cm H_2_O, a white cell count of 92 cells/cu.mm ( >95% mononuclear cells) and no visible organisms. CSF protein was elevated at 90 mg/dL (0.9 g/L), and CSF glucose was normal at 59.5 mg/dL (3.3 mmol/L), compared with a contemporaneous capillary blood glucose of 104.5 mg/dL (5.8 mmol/L). Blood cultures, HIV serology, treponemal serology and enterovirus serology (IgM) were negative. CSF PCR was negative for meningococcus, pneumococcus, enteroviruses and herpesviruses (including HSV1, HSV2, EBV, CMV and VZV). Given the lack of microbiological diagnosis at this point, the CSF was sent for 16S rDNA PCR screening, which identified the rDNA of *Moraxella osloensis*.

Due to ceftriaxone allergy the patient was treated with two weeks of chloramphenicol followed by two weeks of doxycycline to clear residual sinus infection. He made an excellent recovery, being well at three month follow up.

## Discussion

*Moraxella osloensis* is a rare cause of bacterial meningitis, with only a few reported cases. This case illustrates that sinusitis, while a common condition, when severe can predispose to intracranial infection with atypical and low virulence organisms such as *Moraxella* species, which do not commonly cause invasive CNS disease. The cribriform plates of the ethmoid bone form the roof of the nasal cavity, with the ethmoid sinuses lying immediately inferolaterally, and the frontal sinuses and sphenoid sinuses in close proximity anteriorly and posteriorly, respectively. In this case, bony erosion due to extensive sinusitis created a portal of entry for infection in the form of a defect in the cribriform plate, leading to *Moraxella osloensis* meningitis. The degree of loculated sinus inflammation seen in this case is a rarely seen phenomenon in a patient with no preceding symptoms. Of note, the significance of the lymphocytic pleocytosis observed in the CSF of this case is unclear: in other reported cases of *Moraxella osloensis* meningitis a neutrophilic pleocytosis was seen [9], as would be expected in a bacterial meningitis.

This case also demonstrates the diagnostic utility of 16S rDNA PCR screening when microscopy and culture fails to yield a diagnosis. This is an important point as 16S technology is frequently used and rarely detects a causative organism. In our case, as with previous cases [9], we were only able to identify the organism using 16S rDNA PCR screening.

## Conflict of interest statement

The authors declare no conflict of interest.
